# Visual attention mediates the relationship between body satisfaction and susceptibility to the body size adaptation effect

**DOI:** 10.1371/journal.pone.0189855

**Published:** 2018-01-31

**Authors:** Ian D. Stephen, Daniel Sturman, Richard J. Stevenson, Jonathan Mond, Kevin R. Brooks

**Affiliations:** 1 Department of Psychology, Macquarie University, Sydney, Australia; 2 Perception in Action Research Centre, Macquarie University, Sydney, Australia; 3 ARC Centre of Excellence in Cognition and its Disorders, Macquarie University, Sydney, Australia; 4 Centre for Rural Health, University of Tasmania, Launceston, Australia; 5 Centre for Health Research, Western Sydney University, Sydney, Australia; Universita degli Studi di Udine, ITALY

## Abstract

Body size misperception–the belief that one is larger or smaller than reality–affects a large and growing segment of the population. Recently, studies have shown that exposure to extreme body stimuli results in a shift in the point of subjective normality, suggesting that visual adaptation may be a mechanism by which body size misperception occurs. Yet, despite being exposed to a similar set of bodies, some individuals within a given geographical area will develop body size misperception and others will not. The reason for these individual difference is currently unknown. One possible explanation stems from the observation that women with lower levels of body satisfaction have been found to pay more attention to images of thin bodies. However, while attention has been shown to enhance visual adaptation effects in low (e.g. rotational and linear motion) and high level stimuli (e.g., facial gender), it is not known whether this effect exists in visual adaptation to body size. Here, we test the hypothesis that there is an indirect effect of body satisfaction on the direction and magnitude of the body fat adaptation effect, mediated via visual attention (i.e., selectively attending to images of thin over fat bodies or vice versa). Significant mediation effects were found in both men and women, suggesting that observers’ level of body satisfaction may influence selective visual attention to thin or fat bodies, which in turn influences the magnitude and direction of visual adaptation to body size. This may provide a potential mechanism by which some individuals develop body size misperception–a risk factor for eating disorders, compulsive exercise behaviour and steroid abuse–while others do not.

## Introduction

Body size misperception–seeing one’s body as larger or as smaller than it really is–affects a large and growing segment of the population [1–5], with some estimates suggesting that as many as half of adolescents and young adults significantly over- or underestimate their body size [1,2,4–6].

Exposure to extreme body types has been found to be associated with body size and shape misperception (BSSM). Overweight and obese youth with greater exposure to overweight and obese schoolmates and parents are significantly more likely to underestimate their own weight [7], while greater exposure to media is associated with weight overestimation in girls and underestimation in boys, in line with the overrepresentation of idealised thin female bodies and muscular male bodies in media [8]. This effect may be exacerbated in obese individuals by the fact that the weight of obese bodies is typically underestimated (contraction bias), and that changes in body weight become harder to detect as body size increases (Weber’s law) [9].

Recently, authors have noted that the link between exposure to extreme bodies and misperception of own body size resembles a visual adaptation effect [10]. Visual adaptation is a phenomenon that has been known for many decades, perhaps even as far back as Aristotle [11]. When an observer is exposed to an “adaptation” stimulus, for example a red object, the neural channel representing redness adapts, becoming less responsive, and adjusting the observer’s point of subjective neutrality (i.e. the stimulus that appears grey) towards the adapting stimulus (red). Thus, an objectively neutral stimulus (e.g., a white wall) appears opposite to the adapting stimulus (green) [12]. Similar aftereffects have been found in a range of low level stimulus characteristics (i.e. properties that are processed early in the visual system), including motion, orientation and colour [13].

Such visual aftereffects have also been found in higher level properties of visual stimuli, such as the gender, ethnicity [14] and spatial distribution of features [15–17] of human faces. In bodies, visual aftereffects have been found in representations of gender [18] and identity [19], as well as in the perception of size and shape. Exposure to thin (fat) body images has been found to result in average sized bodies appearing fatter (thinner) [20–23]. These effects have been found to transfer across identities, such that adaptation to thin (fat) other-identity bodies causes images of the observer’s own body to appear fatter (thinner) than they are, suggesting that visual adaptation may indeed underlie body size misperception [22,24].

Exposure to rounder body images has been found to increase the liking judgements of round bodies [25,26] in nonclinical participants and the liking of thin bodies in *anorexia nervosa* (AN) patients [26]. Exposure to fat body images has been found to increase the BMI perceived as most attractive in women’s bodies in a nonclinical population [27].

Attentional enhancements of visual adaptation have been demonstrated in a range of different contexts. In the tilt adaptation effect, exposure to an adaptation grating tilted in one direction away from vertical causes a subsequently presented vertical grating to appear tilted in the opposite direction. By instructing participants to preferentially attend to one of two simultaneously presented tilted adaptation gratings, Spivey and Spirn [28] found that the magnitude of the direct tilt adaptation effect was larger in the attended region of the visual field. Similarly, attention directed to one of two simultaneously presented sets of stimuli moving in opposite directions causes an adaptation effect in which a subsequently presented stationary stimulus appears to be moving in the opposite direction to the adaptation stimulus attended [29], while distracting attention from the motion adaptation stimuli by asking participants to simultaneously perform a difficult vowel detection task reduced the magnitude of the motion aftereffect [30]. Further, in a contrast adaptation study, attention to the adapting stimulus has been shown to increase the magnitude of visual adaptation aftereffects [31], which they suggest may be accompanied by an increase neural activation.

However, the impact of visual attention on face and body aftereffects is less well understood. Rhodes et al. [32] found that directing attention towards adaptation face stimuli using a change detection task and a “snap task” (in which participants had to detect immediate repeats of the stimuli), caused increased adaptation to figural distortions of faces. In contrast, attending to the race (or gender) of adaptation face stimuli has been found not to enhance race aftereffects relative to a control [33]. Further, attending to the fatness of bodies has been found to be ineffective in enhancing the strength of adaptation to fat/thin bodies aftereffect relative to a control condition in which participants rated the sexual dimorphism of bodies [34].

The magnitude of body size adaptation effects has been found to be associated with body satisfaction–the extent to which one has a positive subjective evaluation of the weight and shape of one’s own body–women with lower body satisfaction showed reduced adaptation to fat body images [21]. However, the mechanism behind these effects is not known. One proposed explanation for this difference is that the level of body satisfaction influences the degree of attention paid to bodies of different shapes and sizes [34]. Previous studies have shown such relationships. Women with restrained eating patterns have been found to preferentially attend to, and remember, thin body images [35,36]. Similarly, men with low levels of body satisfaction preferentially attended to muscular bodies [36].

In the current study, we investigated the relationship between a) levels of body satisfaction, b) the time spent attending to thin vs fat body adaptation stimuli and c) the magnitude and direction of the resultant body size adaptation effect. Participants were exposed to fat and thin adaptation bodies simultaneously. An eye tracker was used to determine the frequency and duration of fixations on fat and thin bodies (as a measure of visual attention), while the change in point of subjective normality (ΔPSN) caused by exposure to the images was measured. Participants also completed a body satisfaction measure. We hypothesised that individuals with lower levels of body satisfaction would show greater adaptation to thin bodies, and that this relationship may be mediated by the tendency to fixate bodies that are high or low in fat.

## Methods

All work was approved by the Macquarie University Human Research Ethics Committee. All participants gave prior, informed consent in writing.

### Participants

One hundred and ninety-two Caucasian photographic subjects (64 male; age 18–30) were recruited via the Department of Psychology participant pool, and via friends and family of the researchers. Sixty-seven different Caucasian participants (“observers”) (32 male; mean age = 20.85, S.D. = 3.85) were recruited to make perceptual judgements.

### Stimulus production

Photographs were taken of subjects wearing standardised tight-fitting grey singlets and shorts. Subjects faced the camera, and adopted standard anatomical position and a neutral facial expression [24]. Photographs were taken inside a booth that was painted Munsell N5 neutral grey, and illuminated with 15 high accuracy Philips d65 fluorescent tubes in high frequency fixtures to reduce the effects of flicker. A Canon 50D digital camera was used, with exposure, ISO and custom white balance held constant.

A Tanita SC 330 body composition analyser was used to measure weight, body fat and muscle mass, using the Bioelectrical Impedance method, which has an accuracy of ± 2%, and gives readings up to a precision of 0.1 kg [37]. A fixed measuring tape was used to measure height.

**Fig 1:**
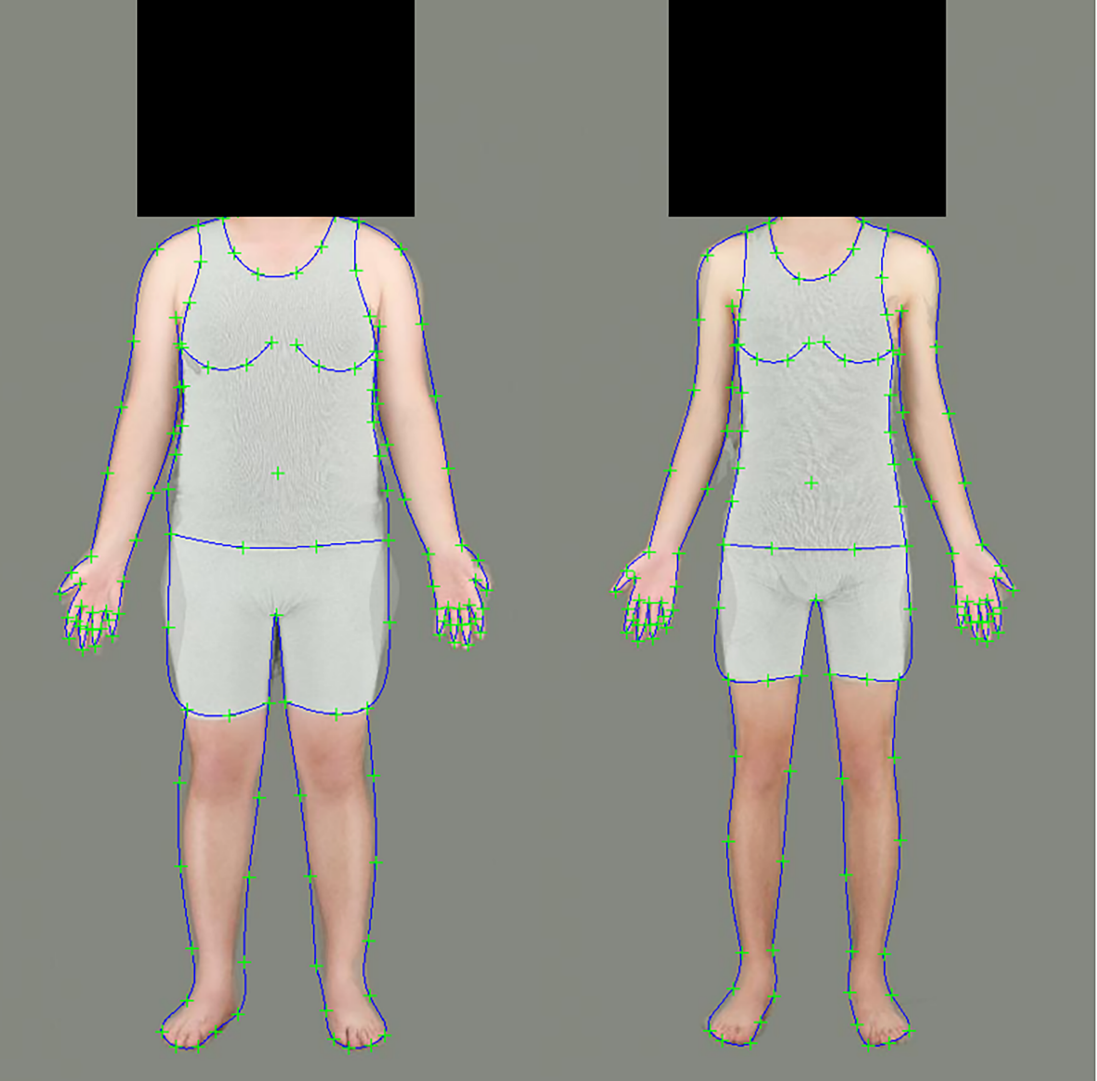
Male high (left) and low (right) fat prototype images used as endpoints for the body fat transforms, showing the locations of the landmark points.

For each image, 130 landmark points were marked to delineate the features of the body using Psychomorph ([38]; Fig 1), and bodies were aligned to remove translational and rotational variation between images.

Stimuli were then manipulated independently in terms of their apparent body fat mass using Psychomorph [23,39]. Images of male and female subjects were treated separately. Since muscle mass and fat mass are highly correlated (due to larger people having more fat and more muscle), regression analysis was used to calculate a fat score for each body, controlling for muscle and height. Muscle mass (kg) and height (cm) were used as predictor variables in a regression model predicting fat mass (kg). The unstandardised residuals of this regression model were saved, providing a body fatness score for each body, controlling for muscle mass and height. The images were then ranked by these body fatness scores. The 10 highest ranked images for each sex were selected, and the location of each landmark point was averaged to create a ‘high fat’ prototype for each sex, while the lowest 10 were averaged to create a ‘low fat’ prototype for each sex (Fig 1). The difference in mean body fat and muscle mass for each prototype pair can be seen in Table 1. Independent samples t-tests showed significant differences in fat mass, but not muscle mass, for the bodies used to create the high and low fat prototypes. These prototype images allowed us to define the image transformations that accompany increases or decreases in body fat independently of any other aspects of the image, using Psychomorph.

**Table 1:** Comparison of fat and muscle mass of the identities that made up the high and low fat prototypes.

Prototype	Fat difference (kg)	Muscle difference (kg)
High fat vs low fat male	11.8*	2.6
High fat vs low fat female	12.0**	-1.5

*p < .05

**p < .01

Twenty-five male and 25 female images with both body fat and muscle mass scores less than 1 standard deviation from the mean, and that were not used in the creation of the prototypes, were selected as test identities (mean BMI = 22.48, *SD* = 2.32). Each image was manipulated in 13 equidistant steps along the body fat dimension from a manipulation of 100% of the difference between the prototypes towards the low fat prototype and ending with a 100% manipulation towards the high fat prototype. This technique allows us to simulate the changes in the average male and female body associated with increases and decreases in fat mass at all 130 landmarks simultaneously, giving a more realistic transform than is achieved using simple geometric transforms such as a uniform widening [40].

Images were formatted to 600 × 900 pixels (15.24 x 21.49 degrees of visual angle when presented on the 17” screen at a viewing distance of 60cm and resolution of 1280 x 1024) for use as test stimuli and 450 × 675 pixels (11.43 x 16.12 degrees) for use as adaptation stimuli to minimise the potential effects of low-level retinotopic adaptation [32]. Test stimuli were presented with a single body on the screen at any one time. Adaptation stimuli were presented in same-identity pairs, with the high fatness version and low fatness version on either side of the screen (i.e. participants would see two images of the same identity simultaneously–one with apparent body fatness increased by 100% of the difference between the prototypes, and one with apparent body fatness reduced by 100% of the difference between the prototypes). Participants were given no directions regarding which image to attend to. By tracking participants’ eye movements while they were presented with increased and decreased body fatness adaptation simultaneously, we were able to measure what proportion of visual attention each participant chose to direct towards thin and fat bodies.

The background of all images was rendered a uniform grey, and the face of each image was obscured with a black square.

### Procedure

Observers provided their age and sex via Qualtrics. While a number of validated questionnaires exist for the measurement of body satisfaction, we are aware of none that fulfilled the requirements of the current study, namely we needed a scale that 1. is for use in non-clinical populations, and 2. is for use with both male and female participants. In line with a number of other studies [41–43], we therefore opted for a single-item measure for the current study: “overall, how satisfied are you with your body?”, which was answered on a seven point Likert-type scale, anchored at 1 (“very dissatisfied”) and 7 (“very satisfied”). Single-item measures have been shown to be highly correlated with a range of validated body satisfaction inventories [44].

Observers were then seated approximately 60cm in front of the 17” TFT screen of a Tobii T120 eye tracker with a screen resolution of 1280 x 1024. Eye tracking was performed at a sampling rate of 120 Hz with high accuracy (0.5°) and drift compensation (less than 0.3°). After an initial calibration task (which involved following a red dot around the screen with one’s eyes), the experiment commenced using the method of adjustment controlled by an app developed in Matlab. Observers saw only stimuli of their own sex in three experimental phases. For each observer, five body identities were randomly selected as practice identities, 10 identities were randomly selected as test stimuli and the remaining 10 identities were used as the adaptation stimuli. The practice phase presented observers with a series of 5 practice bodies (one at a time, in randomised order). Moving the mouse horizontally cycled through the series of 13 frames of the body identity’s fat manipulation. Observers were informed that moving the mouse horizontally would change the appearance of the body (but were not told that it was a fat manipulation) and were asked to “make the body look as normal as possible”, then click to save the data and move onto the next image. Then, in the baseline phase, observers were presented with a sequence of 10 “test” bodies, one at a time in a randomised order, and asked to make them appear as normal as possible. The average amount of fat chosen was taken as the baseline point of subjective normality (PSN).

**Fig 2:**
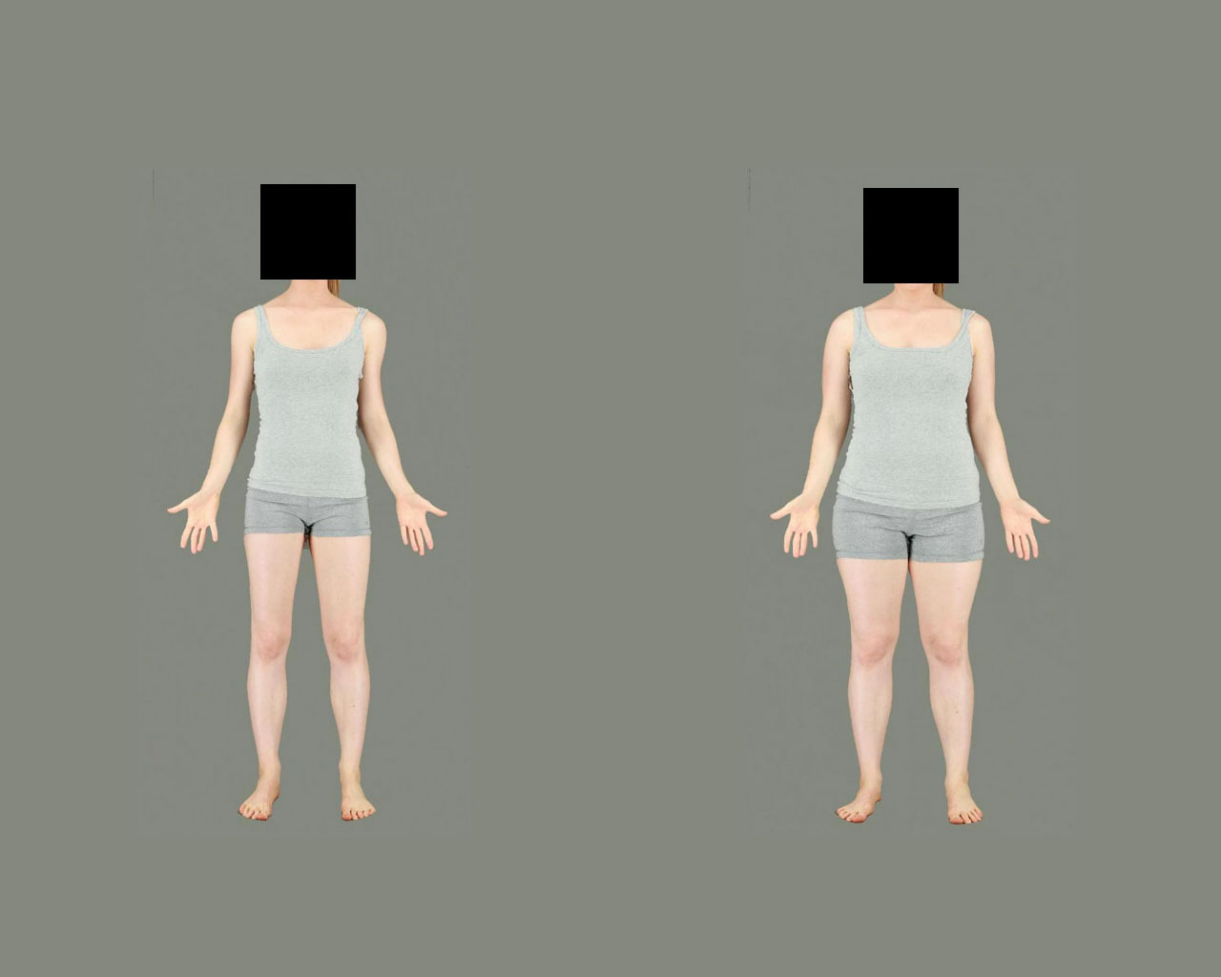
Example of a pair of adaptation bodies.

In the adaptation phase, participants were first shown an initial adaptation sequence involving a series of 10 pairs of same-identity bodies, consisting of one body manipulated to appear high in fat, and one body manipulated to appear low in fat (Fig 2). Each pair was presented twice, once with the low fat body on the left of the screen and once with the low fat body on the right side of the screen, in a random order. Each pair remained on the screen for 6 seconds, to give a total time of 120 seconds. Observers were asked simply to watch the screen, and were not required to make a response. Thereafter, participants were presented with a sequence of the same 10 test stimuli used in the baseline phase, and were again asked to “make the body as normal as possible” using the mouse when a single test stimulus body was presented. These were interspersed with one “top-up” pair of adaptation bodies (selected at random from the set of 10 adaptation stimuli) following each trial, displayed for 6 seconds each, which observers were asked to simply observe without making a response.

### Data processing and analysis

To determine whether participants showed a “normality bias”, the mean frame chosen as appearing most normal in the baseline phase was calculated. For each body, the original frame was number 6, with the lowest fat frame number 0 and highest fat frame number 12. For male trials, the mean frame chosen was 5.59 (SD = 1.53, range 2.8 to 9.3), while for female bodies, the mean frame chosen was 5.46 (SD = 2.09, range 0.9 to 9.3), showing that participants chose a range of levels of transform as appearing most normal, and suggesting that participants were not perceiving the transformed bodies as distorted.

Baseline PSNs were calculated for each observer as the mean fat mass chosen as normal across the trials in the baseline phase. Adaptation PSNs were calculated for each observer as the mean fat mass chosen as normal across the trials in the adaptation phase. Change in PSN (ΔPSN) was then calculated by subtracting the baseline PSN from the adaptation PSN, such that a positive (negative) ΔPSN indicated that an observer’s perception of normality had become fatter (thinner) as a result of adaptation.

Eye tracking data were processed using Eyetracker Output Utility [45] and the iLab utility (v3.6.9) running in Matlab [46]. Following the recommended settings for the iLab utility, a fixation was defined as two or more consecutive samples falling within a 2.55 degree radius, with a minimum fixation duration of 100 ms [46]. Areas of interest (AOIs) were drawn around each body on the adaptation slides, and the total number of fixations and total durations of fixations falling within the fat and thin AOIs were extracted. The percentage of fixations and percentage of fixation duration falling within the thin AOI was calculated for each observer. While it is possible to direct attention to a non-fixated part of the visual field [28], visual fixations are generally considered to be closely associated with the orienting of visual attention in normal circumstances [47–49] and are regularly used as a proxy for visual attention [50,51]

**Fig 3:**
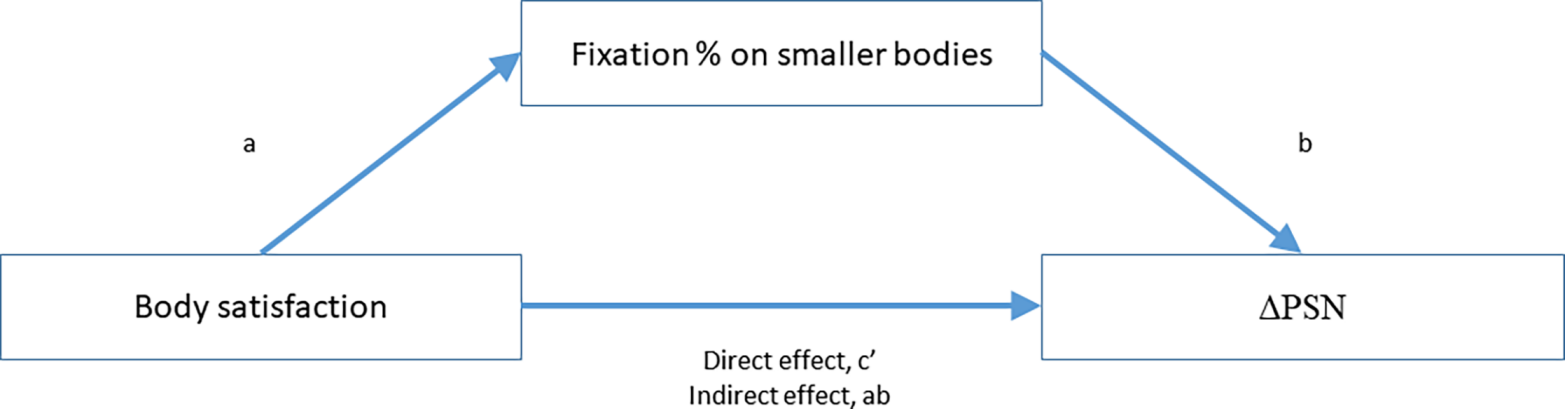
Mediation model for the effect of overall body satisfaction on ΔPSN through percentage of fixation (count or duration) on the thinner bodies. Values can be seen in Table 2.

The PROCESS plugin for SPSS v2.16.3 [52] was used to perform mediation analysis, with ΔPSN as the outcome variable, overall body satisfaction score as the predictor variable, and percentage of thin fixations or percentage of thin fixation duration as the mediating variable (Fig 3). Bootstrapped confidence intervals are reported since this method provides a robust estimate of confidence intervals, regardless of sample size or deviations from normality of the sampling distribution [52].

## Results

Female observers directed a larger percentage of fixations (Male *M* = 49.03%, *SD* = 10.30; Female *M* = 58.98%, *SD* = 15.38) and fixation duration (Male *M* = 49.16%, *SD* = 11.06; Female *M* = 59.16%, *SD* = 16.11) towards the thin bodies than did male observers. No significant difference in overall body satisfaction, *t*(62.78), *p* = .937, was found between female (*M* = 4.00, *SD* = 1.83) and male observers (*M* = 3.97, *SD* = 1.38). While female observers directed significantly more fixations (mean fixation % on thin bodies = 58.98, *SD* = 15.38), *t*(34) = 3.45, *p* = .002, and for significantly longer duration (*M* = 59.16, *SD* = 16.11), *t*(34) = 3.37, *p* = .002, to the thin bodies than would be expected by chance, male observers did not, (fixations *M* = 49.03, *SD* = 10.30), *t*(31) = -.53, *p* = .599, (duration, *M* = 49.16, *SD* = 11.06.), *t*(31) = -.43, *p* = .672.

In the mediation analysis, the effect of observers’ overall body satisfaction on fixation duration (a) was significant, *b* = -.3.09, *p* = .005, indicating that for each point lower on the body satisfaction scale, observers directed around 3% more of their fixation time towards the thin bodies. The effect of fixation duration on ΔPSN (b) was significant, *b* = -.08, p < .001, indicating that observers who directed more of their fixation time towards the thin bodies showed a more negative ΔPSN (their PSN became approximately 85g of body fat thinner post-adaptation for each additional percentage point of fixation time directed towards the thinner bodies). The direct effect of overall body satisfaction on ΔPSN (c’) was not significant, *b* = .18, *p* = .226, while the indirect effect of observers’ body satisfaction on ΔPSN through fixation duration (ab) was significant, *b* = .26, BCa CI [.07, .56], Effect size_cs_ = .19, BCa CI [.05, .35], such that observers who were less satisfied with their bodies tended to fixate for longer on the thin than on the fat bodies, and thus demonstrated a more negative ΔPSN (for each point lower body satisfaction, their PSN became approximately 260g of body fat thinner post-adaptation). This represents a medium effect size [53,54]. This pattern of results was similar when using the fixation count measure in place of fixation duration, and when examining male and female observers separately (Table 2). The pattern of results also remained unchanged when BMI was added to the model as a second mediator (S1 Fig and S1 Table).

**Table 2:** Coefficients of the mediation models.

Sex	Fixation	a	b	c’	ab	Effect size_cs_
**All observers**	Count	-2.90** [-4.92, -.87]^#^	-.09*** [-.13, -.06]^#^	.17 [-.12, .46]	.27 [.08, .56]^#^	.20 [.06, .35]^#^
	Duration	-3.09** [-5.21, -.97]^#^	-.08*** [-.12, -.05]^#^	.18 [-.12, .48]	.26 [.07, .56]^#^	.19 [.05, .35]^#^
**Female**	Count	-3.02* [-5.80, -.25]^#^	-.11*** [-.16, -.06]^#^	.35 [-.07, .77]	.32 [.04, .79]^#^	.21 [.03, .44]^#^
	Duration	-3.21* [-6.11, -.31]^#^	-.09*** [-.14, -.04]^#^	.22 [-.07, .82]	.29 [.03, .72]^#^	.19 [.02, .42]^#^
**Male**	Count	-2.74* [-5.33, -.15]^#^	-.10*** [-.14, -.05]^#^	-.26 [-.61, .10]	.26 [.10, .53]^#^	.24 [.09, .49]^#^
	Duration	-2.94* [-5.72, -.16]^#^	-.09*** [-.14, -.05]^#^	-.26 [-.62, .10]	.27 [.09, .53]^#^	.24 [.08, .50]^#^

* *p* < .05

** *p* < .01

*** *p* < .001

^#^ Bootstrapped 95% confidence intervals do not cross 0. See Fig 1 for model design.

To ensure that our data could not be better explained by a model in which fixation has an indirect effect on ΔPSN via body satisfaction, the analyses were rerun in this configuration. Models were non-significant when examining all observers and when examining only male observers. For female observers, models were significant but effect sizes_cs_ were smaller than in the reported configuration (< .09; S2 Fig and S2 Table).

## Discussion

We predicted that there would be a relationship between overall body satisfaction and direction and magnitude of the body fat adaptation effect, mediated through the number and duration of fixations on thin bodies. This prediction was supported. Male and female observers who were less satisfied with their bodies directed a higher number and duration of fixations to the thin adaptation bodies, and exhibited a more negative ΔPSN (i.e. the body fatness that they perceived as normal became thinner after the adaptation phase than it was before).

As predicted, a significant relationship was found between overall body satisfaction and fixations to thin (compared to fat) bodies, with less satisfied observers directing more attention towards the thinner bodies. This is in line with previous findings that that women exhibiting restricted eating behaviours preferentially attend to thin bodies [36,55]. We found significant relationships between visual attention (as measured by fixation number and duration) to thin bodies and the direction and magnitude of the body fat adaptation effect. Previous studies have found that visual attention enhances the magnitude of high level identity [32], but not ethnicity or gender [33] aftereffects in face stimuli.

These effects were found in both male and female observers. Whilst visual adaptation is thought to affect both men and women, there is evidence to suggest that it may occur differently in each sex. Women show greater lateralisation of neural responses in the extrastriate body area (EBA) and fusiform body area (FBA) during body adaptation, with greater responses in the right hemisphere [56]. Further, women tend to report lower body satisfaction than men [1], and women with low body satisfaction tend to desire lower body weight, while men with low body satisfaction tend to desire higher muscularity [57]. However, sex differences were not observed in the current study, with the pattern of results similar across the sexes, and strongly overlapping confidence intervals of the indirect effects for male and female participants.

Visual adaptation is thought to be the mechanism through which perceptual norms are established in everyday life [58]. Recently, it has been suggested that the body size adaptation effect may provide a mechanism by which body size misperception develops in the real world [10]. This suggestion is bolstered by evidence that body size adaptation effects can transfer from other- to own-identity body images [24]. Future research should seek to establish whether the relationships between body satisfaction, visual attention towards thin bodies, and the body fat adaptation effect exist outside the laboratory, when individuals are navigating more naturalistic environments, and in clinical populations. Further, while there is some evidence that adaptation effects in face stimuli may be long lasting, with durations lasting up to 24 hours [59], little is known about the time course of the body size adaptation effect. This is an important question in determining whether the body size adaptation effect may explain body size misperception in the real world.

Body size misperception has a number of health implications. Individuals who are overweight or obese, and yet underestimate their body size, may be less motivated to lose weight, particularly since changes in body size are more difficult to perceive in larger bodies [9], and may therefore be at higher risk of obesity-related illness, such as type 2 diabetes, heart disease and stroke [4,6,60]. In contrast, normal or underweight individuals who overestimate their body size may be at increased risk of body dissatisfaction and, as a result, increased risk of negative affect and eating disorders [61–64]. Men who perceive themselves to be lower in muscle than they are in reality may be at increased risk of body dissatisfaction, low mood, compulsive exercise behaviour and steroid abuse [65,66].

Our findings may therefore have implications for the treatment of clinical populations in which high levels of body size misperception are likely, to be observed, such as individuals with *anorexia nervosa*, *bulimia nervosa* and, perhaps, muscle dysmorphia. Individuals with lower body satisfaction direct more visual attention to thinner bodies and, in turn, experience stronger negative body fat adaptation, leading them to perceive thinner bodies as normal, which may in turn lead to reduced feelings of satisfaction with their own bodies. If replicated outside the laboratory, this might provide an explanation for why some individuals develop body size misperception, while others do not, despite similar exposure to bodies in the environment and in media. It may be that therapeutic treatment to encourage patients to monitor their exposure to thin idealised bodies, and to attend to more normal bodies may be beneficial in changing patients’ perceptions of their own body weight, which is a major barrier to encouraging patients to resume healthy eating behaviour [67].

A number of limitations of the current study should be acknowledged. First, while single-item measures of body satisfaction are commonly used in the literature [41–43], and scores on single-item body satisfaction measures have been shown to correlate strongly with scores on more time consuming validated body satisfaction scales [44], future studies should consider the use of a validated measure of body satisfaction. Second, our results are currently limited to an undergraduate student sample and, while this is a demographic in which low levels of body satisfaction are common, extending to a broader range of demographics would add to the generalisability of the results. Finally, the current study deals only with visual adaptation in the body fat dimension. Since bodies also vary in muscle mass, visual the relationship between visual attention, body satisfaction and visual aftereffects in the body muscle dimension should be considered in future studies.

In conclusion, this study is the first to find that visual attention directed to thin (as opposed to fat) bodies mediates the relationship between body satisfaction and magnitude of the body fat adaptation effect. These results strengthen the suggestion that visual adaptation may underlie body size misperception, and suggests that individual differences in body satisfaction, associated with different levels of visual attention to fat and thin bodies, may provide an explanation for why some people under- or overestimate their own or others’ body sizes. This may have implications for treatment of body size misperception and its associated disorders.

## Supporting information

S1 Fig Design of the supplementary mediation model. (DOCX)

S2 Fig Design of the reversed mediation model. (DOCX)

S1 Table Coefficients of the supplementary mediation model. ^Ϯ^ p < .10 * p < .05 ** p < .01, *** p < .001, ^#^ Bootstrapped 95% confidence intervals do not cross 0. See S1 Fig for model design.
(DOCX)

S2 Table Coefficients of the reversed mediation model. ^Ϯ^ p < .10 * p < .05 ** p < .01, *** p < .001, ^#^ Bootstrapped 95% confidence intervals do not cross 0. See S2 Fig for model design.
(DOCX)
