# Supplementary material for: Visual attention mediates the relationship between body satisfaction and susceptibility to the body size adaptation effect
Source: PLoS One. 2018 Jan 31;13(1):e0189855. doi: 10.1371/journal.pone.0189855 (PMC5791942; doi:10.1371/journal.pone.0189855)
Supplement: S1 Fig — (DOCX) [file pone.0189855.s001.docx]

Body satisfaction

Fixation % on smaller bodies

ΔPSN

Direct effect, c’

Indirect effect, ab_Total_

a_Fixation_

b_Fixation_

Observer BMI

a_BMI_

b_BMI_

Indirect effect, ab_BMI_

Indirect effect, ab_Fixation_

**S1 Fig: Design of the supplementary mediation model**
