# Supplementary material for: Visual attention mediates the relationship between body satisfaction and susceptibility to the body size adaptation effect
Source: PLoS One. 2018 Jan 31;13(1):e0189855. doi: 10.1371/journal.pone.0189855 (PMC5791942; doi:10.1371/journal.pone.0189855)
Supplement: S2 Fig — (DOCX) [file pone.0189855.s002.docx]

Fixation % on smaller bodies

Body satisfaction

ΔPSN

Direct effect, c’

Indirect effect, ab

a

b

**S2 Fig: Design of the reversed mediation model**
