# Supplementary material for: Visual attention mediates the relationship between body satisfaction and susceptibility to the body size adaptation effect
Source: PLoS One. 2018 Jan 31;13(1):e0189855. doi: 10.1371/journal.pone.0189855 (PMC5791942; doi:10.1371/journal.pone.0189855)
Supplement: S1 Table — Ϯ p < .10 * p < .05 ** p < .01, *** p < .001, # Bootstrapped 95% confidence intervals do not cross 0. See S1 Fig for model design. (DOCX) [file pone.0189855.s003.docx]

| **Sex** | **Fixation** | **a_Fixation_** | **a_BMI_** | **b_Fixation_** | **b_BMI_** | **c’** | **ab_Fixation_** | **ab_BMI_** | **ab_Total_** | **Effect size_cs_ (Fixation)** | **Effect size_cs_ (BMI)** | **Effect size_cs_ (Total)** |
| --- | --- | --- | --- | --- | --- | --- | --- | --- | --- | --- | --- | --- |
| **All observers** | Count | -.03** | -.48 ^Ϯ^ | -9.47*** | .00 | .17 | .27 [.08, .58]# | -.00 [-.07, .06] | .27 [.06, .58]# | .20 [.06, .35]# | -.00 [-.05, .04] | .20 [.04, .36]# |
|  | Duration | -.03* | -.48 ^Ϯ^ | -8.47*** | -.00 | .18 | .12 [.03, .22]# | .00 [-.06, .06] | .26 [.05, .54]# | .19 [.06, .36]# | .00 [-.04, .05] | .19 [.04, .36]# |
| **Female** | Count | -.03* | -.72^Ϯ^ | -10.72*** | .05 | .38 ^Ϯ^ | .32 [.03, .82]# | -.04 [-.22, .07] | .29 [-.06, .80] | .21 [.02, .44]# | -.03 [-.16, .04] | .19 [-.06, .44] |
|  | Duration | -.03* | -.72 ^Ϯ^ | -9.24** | .05 | .41 ^Ϯ^ | .30 [.03, .71]# | -.04 [-.25, .06] | .26 [-.06, .70] | .19 [.02, .43]# | -.03 [-.17, .04] | .17 [-.05, .42] |
| **Male** | Count | -.03* | -.01 | -9.62*** | .00 | -.26 | .26 [.08, .53]# | .00 [-.05, .06] | .26 [.09, .55]# | .24 [.07, .49]# | .00 [-.04, .06] | .24 [.07, .50]# |
|  | Duration | -.03* | -.01 | -9.09*** | -.02 | -.26 | .27 [.09, .53]# | .00 [-.06, .07] | .27 [.10, .55]# | .24 [.06, .49] | .00 [-.05, .06] | .24 [.07, .51]# |

**S1 Table: Coefficients of the supplementary mediation model.** ^Ϯ^ p < .10 * p < .05 ** p < .01, *** p < .001, ^#^ Bootstrapped 95% confidence intervals do not cross 0. See S1 Fig for model design.
