# Supplementary material for: Visual attention mediates the relationship between body satisfaction and susceptibility to the body size adaptation effect
Source: PLoS One. 2018 Jan 31;13(1):e0189855. doi: 10.1371/journal.pone.0189855 (PMC5791942; doi:10.1371/journal.pone.0189855)
Supplement: S2 Table — Ϯ p < .10 * p < .05 ** p < .01, *** p < .001, # Bootstrapped 95% confidence intervals do not cross 0. See S2 Fig for model design. (DOCX) [file pone.0189855.s004.docx]

| **Sex** | **Fixation** | **a** | **b** | **c’** | **ab** | **Effect size_cs_** |
| --- | --- | --- | --- | --- | --- | --- |
| **All observers** | Count | -.04*  [-.07, -.01]^#^ | .17  [-.12, .46] | -.09***  [-.13, -.06]^#^ | -.01  [-.02, .00] | -.04  [-.12, .01] |
|  | Duration | -.04*  [-.06, -.01]^#^ | .18  [-.12, .48] | -.08***  [-.12, .05]^#^ | -.01  [-.02, .00] | -.04  [-.12, .01] |
| **Female** | Count | -.04*  [-.08, -.00]^#^ | -.35  [-.07, .77] | -.11***  [-.16, .06]^#^ | -.02  [-.04, -.00]^#^ | -.08  [-.21, -.01]^#^ |
|  | Duration | -.04*  [-.08, -.00]^#^ | .38^Ϯ^  [-.07, .82] | -.09***  [-.14, -.04]^#^ | -.02  [-.05, -.00]^#^ | -.09  [-.24, -.01]^#^ |
| **Male** | Count | -.05*  [-.10, -.00]^#^ | -.26  [-.61, .10] | -.10***  [-.14, -.05]^#^ | .01  [-.01, .05] | .08  [-.05, .31] |
|  | Duration | -.05*  [-.09, -.00]^#^ | -.26  [-.62, .10] | -.09***  [-.14, .05] | .01  [-.01, .04] | .09  [-.05, .32] |

**S2 Table: Coefficients of the reversed mediation model.** ^Ϯ^ p < .10 * p < .05 ** p < .01, *** p < .001, ^#^ Bootstrapped 95% confidence intervals do not cross 0. See S2 Fig for model design.
